# Detection of the Epstein-Barr Virus and DNA-Topoisomerase II-**α** in Recurrent and Nonrecurrent Giant Cell Lesion of the Jawbones

**DOI:** 10.1155/2013/327424

**Published:** 2013-07-15

**Authors:** Manal M. Zyada, Nagla M. Salama

**Affiliations:** Faculty of Dentistry, Mansoura University, P.O. Box 40, Mansoura 35516, Egypt

## Abstract

The aims of this study were to determine whether the expression of Topo II-*α* correlates with presence of EBV in giant cell lesion of the jawbones and whether it is predictive of clinical biologic behavior of these lesions. Paraffin-embedded tissues from 8 recurrent and 7 nonrecurrent cases of bony GCLs and 9 peripheral giant cell lesions (PGCLs) as a control group were assessed for the expression of EBV and Topo II-*α* using immunohistochemistry. The results showed positive staining for Topo II-*α* in mononuclear stromal cells (MSCs) and multinucleated giant cells (MGCs). Student *t*-test showed that mean Topo II-*α* labelling index (LI) in recurrent cases was significantly higher than that in non-recurrent cases (*P* = 0.0001). Moreover, Spearman's correlation coefficients method showed a significant correlation between DNA Topo II-*α* LI and both of gender and site in these lesions. Moderate EBV expression in relation to the highest Topo II-*α* LI was observed in two cases of GCT. It was concluded that high Topo II-*α* LIs could be identified as reliable predicators for the clinical behavior of GCLs. Moreover, EBV has no etiological role in the benign CGCLs in contrast to its role in the pathogenesis of GCTs.

## 1. Introduction

Central giant cell lesions (CGCLs) of the jaws are relatively uncommon reactive bone disorders in which etiology, pathogenesis, and therapeutic have not been clearly defined [1]. The World Health Organization (WHO) defined this entity as nonneoplastic and localized benign but sometimes aggressive osteolytic proliferation and has a high recurrence rate [2, 3]. In contrast to the CGCL, the true giant cell tumor of the jaws (GCT) is rare and local prognosis is considered worse in GCT than in CGCL [4].

There is a basic question whether CGCG and GCT are separate entities or variants of the same disease. The study of cell cycle-associated proteins in both lesions may give insights into clarifying such question. The expression of these proteins is also important to determine the cell cycle regulation in both tumors.

The topoisomerase II (Topo II) enzymes are required in many aspects of DNA metabolism including replication, transcription, chromosome segregation, and cell proliferation [5]. Because the expression of Topo II-*α* isoform increases during the late S phase, decreases at the end of the M phase, and is dramatically reduced in the G1/G0 phase of the cell cycle [6], an anti-Topo II-*α* antibody labels cells in the S, G2, and M phases of the cell cycle [7]. Two Topo II iso-enzymes, Topo II-*α* and Topo II-*β*, have been characterized in mammalian cells [8]. The expression of Topo II a has been associated with the rate of tumor cell proliferation [9]. 

EBV is a member of the herpes virus family. It is now known that EBV infects 90% of the world's adult population [10]. EBV is an important etiologic factor in a variety of diseases, benign and malignant disorders [11, 12]; virtually little is known about the possible role of viruses and their interactions with genes [13].

Even though the clinical differences and histologic features of GCLs have been well documented, the role of cell cycle-associated topoisomerase II-*α* (DNA-Topo II-*α*) regarding clinical behavior of these lesions and the possible role of EBV in the interaction with this protein remains unclear.

The aims of this study were to determine whether EBV and DNA-Topo II-*α* are present in giant cell lesion (CGCL) of the jaws, whether the expression of Topo II-*α* correlates with clinicopathologic parameters and presence of EBV, and whether they are predictive of clinical biologic behavior of these lesions.

## 2. Materials and Methods

Twenty-four archival biopsies previously diagnosed as giant cell lesions were included in this study. Group I consists of 9 cases of peripheral giant cell granuloma (PGCL) representing the control group. Group II consists of 15 cases of bony giant cell lesions. Of these bony lesions, 8 showed no recurrence (8 cases CGCL); 7 cases showed local recurrence (5 cases CGCL and 2 cases GCT). These cases were obtained from paraffin blocks archives of the Oral and General Pathology Departments, Faculty of Dentistry and Faculty of Medicine, Mansoura University. CGCLs were classified according to* WHO Classification of Head and Neck Tumors *published in July 2005 [2].

## 3. Histological Study

Sections of 4 *μ*m thickness were cut, deparaffinized, rehydrated, and stained with (a) hematoxylin and eosin (H&E) for reevaluation and confirmation of histopathological examination and diagnosis and (b) for the immunohistochemical evaluation of both EBV and Topo II-*α* expression.

## 4. Immunohistochemical Study

Paraffin sections were used for immunostaining for monoclonal antibodies for EBV CS1-4 (Dakopatts, diluted at 1 : 50) that recognizes EBV-encoded LMP1 and mouse anti-human Topo II-*α*  protein (DAKO, clone: Ki-S1, isotype: IgG2a) was used. The bottle contains 1 mL of Topo II-*α* antibody provided in liquid form as purified IgG diluted in 0.05 M Tris/HCL, 15 mM NaN, and pH 7.2, 1% bovine serum albumin (BSA). Bottle number 2 was applied to 1 : 80 dilutions in 1% BSA in phosphate-buffered saline (PBS) by the strept avidin-biotin complex method (Lab Vision Corporation strept avidin-biotin complex universal kit, Ultra Vision Detection System, antipolyvalent, horseradish peroxidase (HRP)/diaminobenzidine (DAB), Fremont, CA, USA) [14]. Positive and negative controls were included. For negative control slide, one vial (3 mL) of nonimmune serum or immunoglobulins in PSA with 0.09% sodium azide was used.

## 5. Staining Assessment

The immunoreactivity of antibodies to EBV was assessed on a visual analogue scale by semiquantifying the nuclear and cytoplasmic staining. Immunoreactivity was scored as either absent (−), low (1+, ≥25% of positive tumor cells), moderate (2+, 26% to 75% of positive tumor cells), or diffuse (3+, ≥75% of positive tumor cells). Topo II-*α* immunoreactivity was assessed in MGCs and MSCs separately by the image analysis software (Image J, 1.29 t, NH, USA). Images were acquired by a high-resolution single-chip charged-coupled device (CCD) video camera in lesional regions with subjectively the highest number of immunoreactive cells. A total of 4 adjacent medium power microscopic fields were analyzed at the power of ×20. Automatic rather than operator-guided color thresholding was adopted to achieve maximum standardization. Computerized calculation of the total surface area of immunoreaction was expressed as a fraction (percentage) of the total surface area of the microscopic field (immunostained area fraction). The LI was defined by the percentage of positively stained cells. Immunostaining for EBV was evaluated on the basis of immunoreactivity.

## 6. Statistical Analysis

The statistical significance of differences in percentages of cases positive for EBV immunostaining was determined by Pearson's chi-square. The percentage of Topo II-*α*-positive cell was tabulated as a mean. Statistical analysis was done using SPSS for Windows. One-way ANOVA (analysis of variance) test and a Student's *t*-test for analysis of means were performed. The Spearman rank correlation analysis was used to analyze the relationship among the indices. A *P*  value < 0.05 indicated statistical significance.

## 7. Results

### 7.1. Immunohistochemical Findings

**Figure 1:**
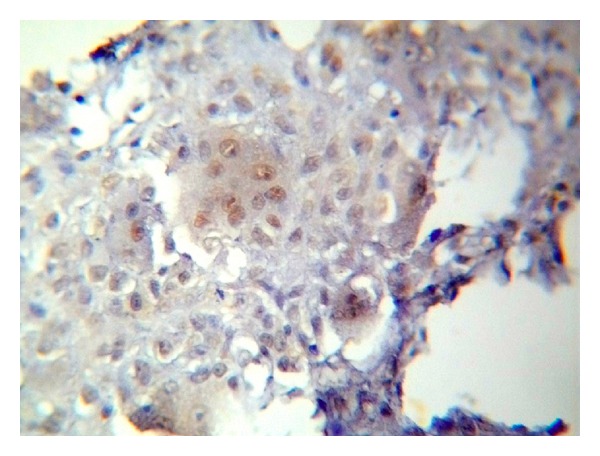
Nuclear and cytoplasmic positive reaction of EBV in both MGCs and MSCs of GCL of jawbones (ABC ×40).

The positive immunohistochemical reactivity to EBV appeared as brown cytoplasmic and nuclear staining reaction mainly in mononuclear stromal cells (MSCs) and in only a few multinucleated giant cells (MGCs) (Figure 1). 

**Figure 2:**
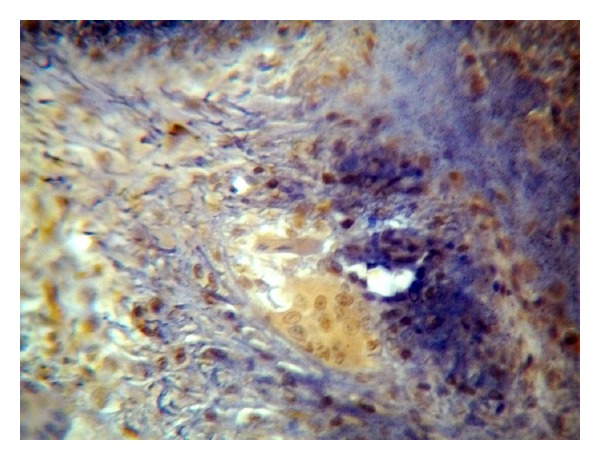
Nonrecurrent GCL case showed nuclear and cytoplasmic Topo II-*α* staining in both MGCs and MSCs (ABC ×20).

**Figure 3:**
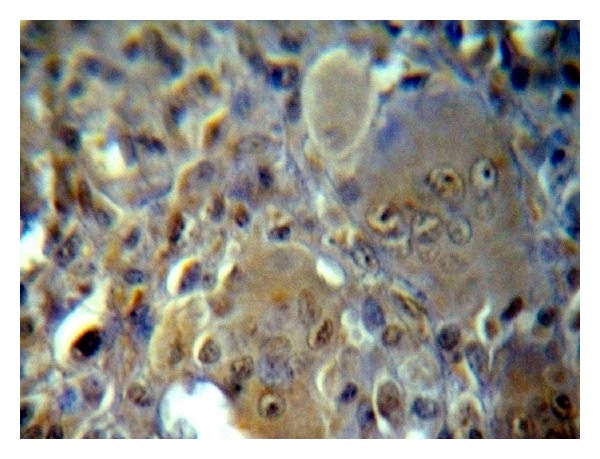
Another case of nonrecurrent GCL showed high Topo II-*α* immunoreactivity in MSCs in comparison with MGCs (ABC ×40).

**Figure 4:**
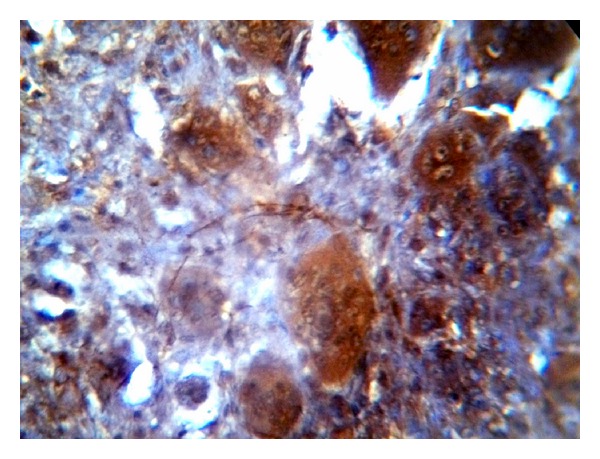
Recurrent GCL cases showed high Topo II-*α* immunoreactivity in both MGCs and MSCs (ABC ×40).

The positive immunohistochemical staining for Topo II-*α* appeared as a brown cytoplasmic and nuclear reaction in MSCs as well as MGCs (Figures 2, 3, and 4).

**Figure 5:**
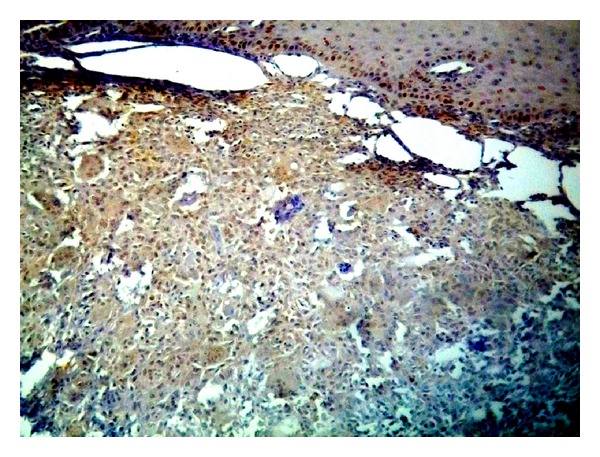
PGCL showed nuclear and cytoplasmic immunoexpression of Topo II-*α* in basal and parabasal layer in epithelial tissue. Also, Topo II-*α* immunoreactivity was observed in MGCs (ABC ×20).

Topo II-*α* was mainly observed in the basal and parabasal cell layers of normal squamous epithelium (Figure 5), while EBV expression showed negative reaction.

Immunohistochemical reactivity for EBV and Topo II-*α* in GCLs is summarized in Table 1

Mean values of Topo II-*α* LI were greater in MSCs than MGCs. Students *t*-test revealed that there was significant difference between PGCL and bony GCLs (*P* < 0.05). One-way ANOVA test showed significant difference between all studied lesions (*P* = 0.0001).

### 7.2. Correlation between Clinicopathological and Immunohistochemical Findings

Students *t*-test provides us with statistical differences between female and male in relation to mean values for Topo II-*α* LI in both MGCs and MSCs (*P* = 0.004, 0.024, resp.). Also, there was a significant difference between mandible and maxilla (*P* > 0.001). However, no significant difference was observed between young and old ages. Also, mean Topo II-*α*-LI in recurrent cases of CGCLs was significantly higher than that in nonrecurrent cases of CGCLs (*P* = 0.0001) (Table 2). Moreover, Spearman's correlation coefficients method showed a significant correlation between DNA-Topo II-*α* LI and both of gender and site in these lesions (*r* = 0.632; *P* = 0.003  *r* = 0.571; *P* = 0.002, resp.).

Although most of all GCL cases showed negative reaction of EBV, two cases of GCT showed moderate EBV expression in relation to the highest Topo II-*α* LI.

## 8. Discussion

Giant cell lesions of the oral cavity are a well-recognized entity; controversies surrounding the relationship between central giant cell lesion of the jaws and giant cell tumor of long bone have revolved around their biologic behavior, histopathologic features, and clinical response to conservative therapy [15].

Human topoisomerase II plays a crucial role in DNA replication and repair. It exists in two isoforms: topoisomerase II-alpha (*α*) and topoisomerase II-beta (*β*). The *α* isoform is localized predominantly in the nucleus, while the *β* isoform exhibits a reticular pattern of distribution both in the cytosol and in the nucleus [16].

In the present work, the intense nuclear and cytoplasmic immunoexpression of Topo II-*α* is observed in basal and parabasal layer in studied epithelial tissues, although Earnshaw et al. [17] revealed that Topo II-*α* has been shown to be a component of two highly insoluble protein fractions from chromosomes and nuclei, so these observation suggested that Topo II *α* might be an integral structure of the nucleus.

In this study, cell proliferation as evaluated by Topo II-*α* immunoreactivity was seen mainly in mononuclear stromal cells (MSCs) and in only a few (multinucleated giant cells) MGCs in two cases of GCT of jaws. These results were supported by some previous data which have shown that stromal cells represent high proliferative activity [18]. Furthermore, many investigators have previously revealed that MSCs are a neoplasmic element of GCT, whereas MGCs are a reactive component [19, 20].

However, Topo II-*α* immunoreactivity was detected in both MGCs and MSCs in all cases of CGCLs. These results were in accordance with de Souza et al. [21] who state that the differences observed in proliferative activity do not explain the different biological behaviors of CGCG and GCT, as reactive lesions may show increased proliferative activity. The authors emphasize that since CGCG and GCT occur in different sites, it is difficult to compare accurately their biological evolution. Nevertheless, de Souza et al. [21] suggest that CGCG and GCT could represent variants of the same disease.

With regard to the Topo II-*α* LI, there was a statistically significant difference observed among recurrent cases compared with non-recurrent ones. This might indicate an intimate relationship between increasing Topo II-*α* LI and the aggressive giant cell lesions. This finding was in agreement with Lee et al. [22] who observed an association between Topo II-*α* LI and the aggressive clinical behavior in thyroid neoplasia.

All our CGCLs expressed Topo II-*α*. A significant relationship between Topo II-*α* LIs and clinical parameters of CGCLs was observed demonstrating an enhanced Topo II-*α* expression in aggressive lesions. For the prediction of the individual prognosis in patients with CGCLs, we have reason to believe that a combination of clinical parameters and Topo II-*α* immunohistological parameters might be helpful for the classification of CGCLs into aggressive and nonaggressive lesions. In our opinion, high Topo II-*α* LIs could be identified as reliable predicators for the clinical behavior of CGCLs.

Our results demonstrated that despite the absence of EBV immunoreactivity in both recurrent and non-recurrent cases of CGCLs, all recurrent GCT cases revealed positive nuclear and cytoplasmic reactions of EBV mainly in MSCs and in only a few MGCs in relation to the high Topo II-*α* LIs. These findings explain that EBV has no etiological role in the benign CGCLs in contrast to its role in the pathogenesis of GCTs. 

In summary, the current study showed that EBV expression was concordant with that of Topo II-*α* LIs in GCT of jaws. These results suggest that EBV and Topo II-*α* may play a crucial role in cell proliferation of this tumor. Taken together, our results may provide a possible link between presence of EBV and cell cycle control. In addition, Topo II-*α* LIs may be a useful indicator of cell proliferation in MSCs of this tumor.

## 9. Conclusions

Our findings show that recurrent cases of GCLs of the jaws have a higher Topo II-*α* LIs compared with those of the non-recurrent ones. Thus, these findings suggested that high Topo II-*α* LIs could be identified as reliable predicators for the clinical behavior of GCLs. Moreover, our results revealed that EBV has no etiological role in the benign CGCLs in contrast to its role in the pathogenesis of GCTs.

These results are preliminary because of the small sample size and should be verified in a larger number of cases. Further research is needed to clarify the pathogenesis and nature of these giant cell lesions and other markers have to be investigated in order to answer the question of whether these lesions represent the development of a single pathologic process or not.

## Figures and Tables

**Table 1:** Immunohistochemical findings of EBV and Topo II-*α* in giant cell lesion of jawbones.

Lesions	No. of cases	EBV				Topo II-*α* LI (%)^a^	Topo II-*α* LI (%)^b^
		−	+	++	+++		
PGCL	9	9	0	0	0	5.70 ± 2.02	16.81 ± 2.64
CGCL	13	12	1	0	0	7.64 ± 0.63	22.90 ± 5.62
GCT	2	0	0	2	0	11.42 ± 4.02	39.22 ± 5.75

MGCs: multinucleated giant cells; MSCs: mononuclear stromal cells.

^a^Mean ± standard deviation of Topo II-*α*in MGCs.

^b^Mean ± standard deviation of Topo II-*α* in MSCs.

**Table 2:** Mean values of Topo II-*α* in relation to clinical parameters in central giant cell lesion of jawbones.

Variable	Total no. (%)	Topo II-*α* ^a^	*P* value	Topo II-*α* ^b^	*P* value
Age (y)					
<30	8 (61.5%)	1.49 ± 0.63	0.273	8.00 ± 7.89	0.716
≥30	5 (38.5%)	1.09 ± 1.01	NS	7.00 ± 6.57	NS
Sex					
Male	3 (23.1%)	0.75 ± 0.17	0.001	8.12 ± 2.77	0.021
Female	10 (76.9%)	1.71 ± 0.87	*S	15.24 ± 4.64	*S
Site					
Maxilla	5 (38.5%)	1.70 ± 0.84	0.001	5.69 ± 2.00	0.001
Mandible	8 (61.5%)	2.50 ± 0.14	*S	8.16 ± 0.88	*S
Clinical behavior					
Nonrecurrent	8 (61.5%)	1.15 ± 0.11	0.002	5.67 ± 2.01	0.001
Recurrent	5 (38.5%)	2.07 ± 0.95	*S	8.17 ± 0.87	*S

MGCs: multinucleated giant cells; MSCs: mononuclear stromal cells.

^a^Mean ± standard deviation of Topo II-*α* in MGCs.

^b^Mean ± standard deviation of Topo II-*α* in MSCs.

*t*-test.

NS: not significant.

*S: significant.
